# Importance of Tricuspid Regurgitation Velocity Threshold in Risk Assessment of Pulmonary Hypertension-Long-Term Outcome of Patients Submitted to Aortic Valve Replacement

**DOI:** 10.3389/fcvm.2021.720643

**Published:** 2021-11-10

**Authors:** Cora Garcia-Ribas, Mirea Ble, Miquel Gómez, Aleksandra Mas-Stachurska, Núria Farré-López, Mercè Cladellas

**Affiliations:** ^1^Department of Cardiology, Hospital del Mar, Barcelona, Spain; ^2^Department of Medicine, Universitat Autònoma de Barcelona, Barcelona, Spain; ^3^IMIM-Hospital del Mar Medical Research Institute, Barcelona, Spain

**Keywords:** pulmonary hypertension, echocardiography, tricuspid regurgitation threshold, aortic stenosis (AS), left heart valve disease

## Abstract

**Background:** The upper physiological threshold for tricuspid regurgitation velocity (TRV) of 2.8 m/s proposed by the Pulmonary Hypertension (PH) guidelines had been questioned. The aim of this study was to evaluate the prognostic significance of preoperative PH in patients with aortic stenosis, long-term after valve replacement, using two different TRV thresholds (2.55 and 2.8 m/s).

**Methods:** Four hundred and forty four patients were included (mean age 73 ± 9 years; 55% male), with a median follow-up of 5.8 years (98% completed). Patients were divided into three PH probability groups according to guidelines (low, intermediate and high) for both thresholds (TRV ≤ 2.8 m/s and TRV ≤ 2.55 m/s), using right atrial area>18 cm^2^ and right ventricle/left ventricle ratio>1 as additional echocardiographic variables.

**Results:** In patients with measurable TRV (*n* = 304), the low group mortality rate was 25% and 30%, respectively for 2.55 and 2.8 m/s TRV thresholds. The intermediate group with TRV > 2.55 m/s was an independent mortality risk factor (HR 2.04; 95% CI: 1.91 to 3.48, *p* = 0.01), in contrast to the intermediate group with TRV>2.8 m/s (HR 1.44; 95% CI: 0.89 to 2.32, *p* = 0.14). Both high probability groups were associated with an increased mortality risk, as compared to their respective low groups. When including all patients (with measurable and non-measurable TRV), both intermediate groups remained independently associated with an increased mortality risk: HR 1.62 (95% CI 1.11 to 2.35 *p* = 0.01) for the new cut-off point; and HR 1.43 (95% CI: 0.96 to 2.13, *p* = 0.07) for guidelines threshold.

**Conclusion:** A TRV threshold of 2.55 m/s, together with right cavities measures, allowed a better risk assessment of patients with PH secondary to severe aortic stenosis, with or without tricuspid regurgitation.

## Introduction

Pulmonary hypertension (PH) is a potential complication of many cardiovascular and respiratory diseases. Transthoracic Doppler echocardiography remains the most important non-invasive screening tool due to its widespread availability and ease of use. The current guidelines from the European Society of Cardiology and the European Respiratory Society (ESC/ERS) (1) recommend grading PH probability based on tricuspid regurgitation velocity (TRV) peak at rest and on additional echocardiographic signs suggestive of PH. PH probability is thus classified as low (TRV ≤2.8 m/s), intermediate (TRV 2.9–3.4 m/s) and high (TRV >3.4 m/s). However, certain authors have suggested that PH estimation may often be misclassified by traditional thresholds (2), and that current cut-off points may need to be reconsidered, since small increases in TRV, even at values considered normal, are independently associated with an increased mortality (3–5). In this regard, an interesting investigation by Marra et al. (6) proposed that TRV cut-off should be lowered to 2.55 m/s in healthy individuals.

PH is an important contributor to morbidity and mortality in patients with aortic stenosis (7). Previous reports showed that a high PH probability was related to an increased morbidity and mortality (8, 9). In these studies, the intermediate probability groups were also associated with worse outcomes, but did not reach significance on multivariate analysis. On another hand, a recent study showed that the incorporation of right cavities measurements as additional echocardiographic signs to TRV, allowed a better prognostic classification of patients with severe aortic stenosis after valve replacement (10). Thus, according to the current ESC/ERS guidelines, the aim of the present study was to evaluate the preoperative PH prognosis of patients with severe aortic stenosis, by using two different TRV thresholds: the 2.8 m/s proposed by current guidelines and a new cut-off point of 2.55 m/s, together with right cavities measurements.

## Methods

### Research Question and Study Design

An observational cohort with severe aortic stenosis undergoing aortic valve replacement was included from January 2005 to June 2018 at the Department of Cardiology of the Hospital del Mar (Barcelona, Spain). The database contains detailed clinical information of risk factors, number of replaced valves, postoperative complications, and an annual follow-up. All patients underwent surgery (aortic valve replacement) at our reference hospital (Hospital de la Santa Creu i Sant Pau). Patients with isolated aortic insufficiency or double valve replacement, and patients submitted to transcatheter aortic valve replacement were excluded.

The study was approved by the institutional review boards at the CEIm-Parc de Salut Mar institution (approval number 2019/8278) and was conducted in accordance with the amended Declaration of Helsinki.

### Endpoints and Definitions

The main aim of this study was to investigate the prognostic significance of preoperative PH secondary to severe aortic stenosis, long-term after aortic valve replacement, using two different TRV thresholds (TRV ≤ 2.55 m/s and TRV ≤ 2.8 m/s), together with right cavities measurements.

In order to assess all-cause mortality following aortic valve replacement, patients were divided into three PH probability groups as proposed by ESC/ERS guidelines (low, intermediate, and high), for both thresholds. Right atrial area (RAA) >18 cm^2^ and right ventricle (RV)/left ventricle ratio >1 were used as additional echocardiographic variables (Table 1). The presence of a single echocardiographic sign did not change the PH probability level.

**Table 1:** Echocardiographic probability of pulmonary hypertension according to current ESC/ERS guidelines and the new cut-off point.

Tricuspid regurgitation velocity peak (m/s) New cut-off value^*^	**Tricuspid regurgitation velocity peak (m/s) Guidelines-2015**	**RAA (end-systole) >18 cm^**2**^ RV/LV basal diameter ratio >1**	**Echocardiographic pulmonary hypertension probability**
≤ 2.55 or not measurable	≤ 2.8 or not measurable	No	Low
≤ 2.55 or not measurable	≤ 2.8 or not measurable	Yes	Intermediate
2.6–3.4	2.9–3.4	No	
2.6–3.4	2.9–3.4	Yes	High
> 3.4	> 3.4	Not required	

* *Marra et al. (6). RAA, right atrial area; RV, right ventricle; RV, left ventricle*.

In a first analysis, only patients with measurable tricuspid regurgitation (TR) were studied. Same analyses were subsequently performed including all patients. All groups were compared to their respective low PH probability group, considered the reference group. Finally, the association between TRV and right cavities measurements was explored.

### Study Protocol

Preoperative variables included classic cardiovascular risk factors, history of myocardial infarction, atrial fibrillation or flutter, stroke, New York Heart Association functional class III or IV, left ejection fraction <50%, chronic obstructive pulmonary disease (COPD) when FEV1 was <70%, or if the diagnosis was previously made by a physician. All patients underwent coronary angiography before surgery and the European System for Cardiac Operative Risk Evaluation score was recorded for each one. Definitions of risk factors have been previously described (11).

### Clinical Follow-Up

After aortic valve replacement, clinical appointments were carried out at discharge, and later on at 3 and 12 months. After this period, follow-up appointments were performed once a year by a cardiologist or when the patient requested it at our institution. All clinical events were recorded in a computerized database.

### Doppler Echocardiographic Methods

A standard 2D echocardiography was performed in all patients before undergoing surgery, in order to evaluate TR degree and velocity using continuous and color Doppler, and to assess RV function by tricuspid annular plane systolic excursion (TAPSE) using the M-mode technique. TAPSE <17 mm was indicative of RV systolic dysfunction (12). Echocardiographic exams were performed with commercially available ultrasound systems (Vivid 7 and E9 GE Healthcare Vingmed, Trodheim) according to the American Society of Echocardiography and the European Association of Cardiovascular Imaging guidelines (12). TRV peak was recorded in multiple views using continuous Doppler to obtain the optimal appearing tricuspid regurgitant jet (most frequently from the apical 4-chamber view). TR severity was graded as mild, moderate, severe or non-measurable (trivial or absent) based on TR jet size by color flow imaging. RAA was measured in the apical four-chamber view at end-systole. RV and left ventricle basal diameter were measured from the apical 4-chamber view obtained at the end-diastole.

### Statistical Analysis

Continuous data are presented as mean ± standard deviation (SD) or median (25–75th percentile range). Categorical variables are shown as frequencies and percentages. Relationships among continuous variable groups were explored using 2-tailed *t-*tests (for normally distributed variables) or Mann-Whitney *U*-tests (for non-normally distributed variables). Chi square tests and Fisher's exact tests were used to examine the association between groups and baseline categorical variables. The association between TVR and right cavities measurements was assessed by Spearman's correlation.

Kaplan-Meier survival curves to time-to-event data were compared with the Log-rank test, taking as reference the low probability group for both thresholds. Multivariate survival analysis using Cox proportional hazards models, adjusted for age, sex and significant variables related to PH and mortality were used to assess the independent association between PH probability and postoperative prognosis for both thresholds. The low PH probability group was considered the reference group in all cases. Results were estimated for all-cause mortality and were presented as hazard ratios (HR) and 95% confidence intervals (CI). Significance was set at a *p* < 0.05. For multivariable models, all parameters associated with death on univariate analysis with *p* < 0.10 were included. Statistical analyses were performed with the R software version 3.4.2.

## Results

### Study Population

During the study period, 444 consecutive patients were included before surgery and were subsequently assessed after aortic valve replacement with a median follow-up of 5.8 years (interquartile range 3.2–8.1 years), with only 2% of the patients lost during follow-up. Mean age was 73 ± 9 years and 55% were male. TRV was detected as non- measurable in 140 patients (32%), mild in 229 patients (52%) and moderate in 75 patients (17%). No severe TR was observed.

Before surgery, patients were divided into three PH probability groups. According to the guidelines thresholds, the following groups were obtained: low (*n* = 256, 58%), intermediate (*n* = 119, 27%) and high (*n* = 69, 15%). When the new TRV threshold of 2.55 m/s was applied, 47 patients from the low group were reclassified to the intermediate group, and nine patients from the intermediate group were relocated into the high group. Thus, with the new cut-off point, the followings groups were observed: low (*n* = 209, 47%), intermediate (*n* = 157, 35%) and high (*n* = 78, 18%).

### Baseline Characteristics and Clinical Outcomes of Patients With Tricuspid Regurgitation

**Figure 1:**
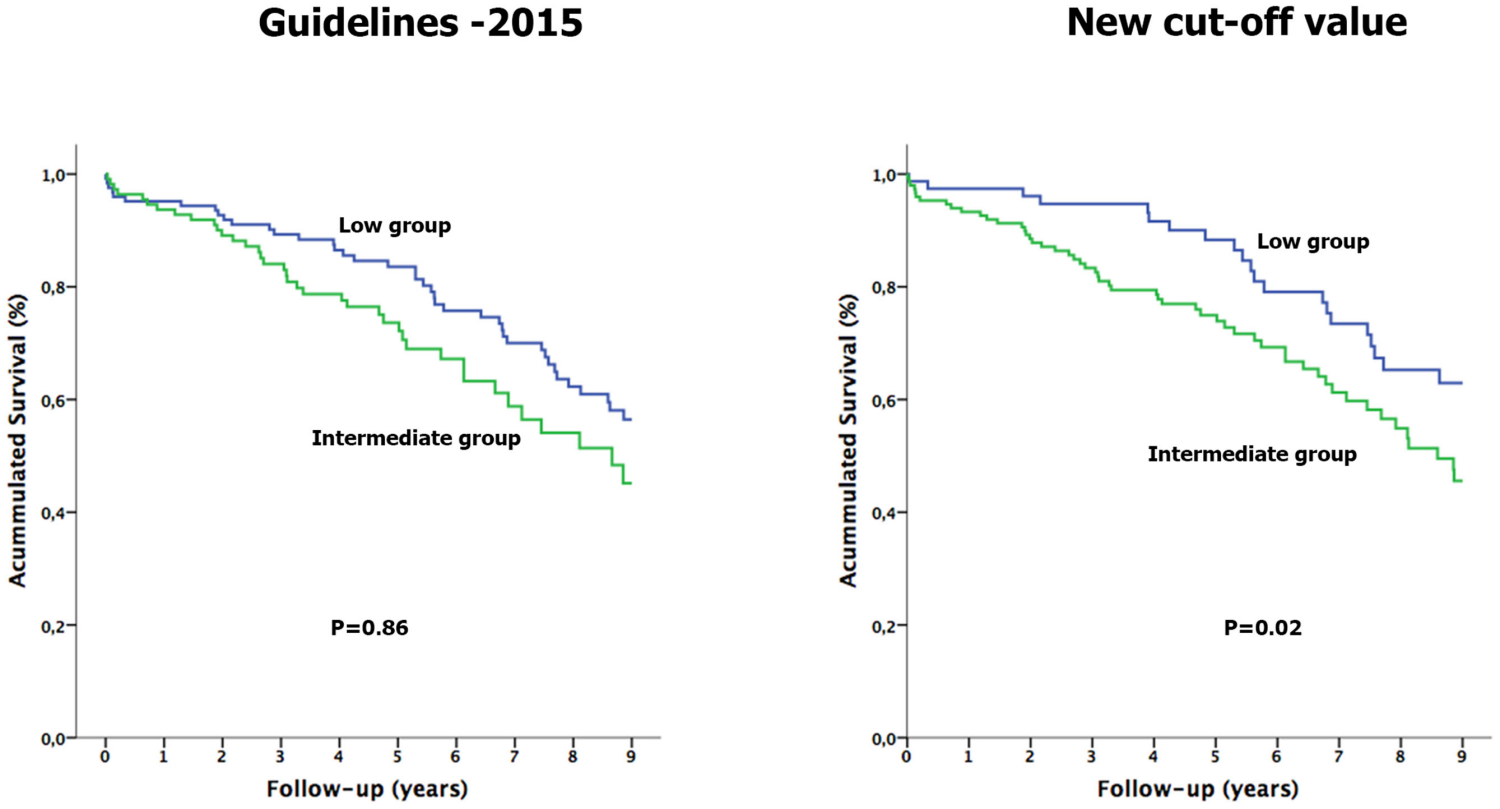
Kaplan-Meier survival curves analysis of patients with tricuspid regurgitation, according to ESC/ERS guidelines and the new cut-off point thresholds.

In the 304 patients with TR, the statistically significant differences observed between the intermediate and high probability groups as compared to their respective low group were similar for both cut-off points (Table 2). Likewise, in the Kaplan Meier curves to time-to-event data, no differences were observed in terms of mortality rate between the three groups, for both cut-off points (Figure 1).

**Table 2:** Baseline characteristics and all-cause mortality during follow-up between groups, according to current ESC/ERS guidelines and the new cut-off point thresholds, in patients with tricuspid regurgitation.

	**ESC/ERS guidelines-2015**			**New cut-off point value**		
	**Low (*n* = 124)**	**Intermediate (*n* = 111)**	**High (*n* = 69)**	**Low (*n* = 77)**	**Intermediate (*n* = 149)**	**High (*n* = 78)**
**Risk factors:**						
Age (*n* ± SD)	72 ± 9^*^	73 ± 8	75 ± 7^*^	71 ± 9^*^	73 ± 9	76 ± 7^*^
Male (*n*, %)	70 (57)^*^	64 (43)^*^	41 (59)	48 (62)^*^	64 (43)^*^	46 (59)
Hypertension (*n*, %)	86 (69)	83 (75)	54 (78)	50 (65)	113 (76)	60 (77)
Diabetes mellitus (*n*, %)	32 (26)	34 (31)	24 (35)	21 (27)	44 (30)	25 (32)
Dyslipidemia (*n*, %)	79 (64)	51 (52)	38 (55)	47 (61)	87 (58)	41 (53)
Current Smoking (*n*, %)	11 (8.9)	12 (11)	6 (8.7)	6 (7.8)	14 (9.4)	9 (12)
NYHA III-IV class (*n*, %)	26 (21)^*^	21 (19)	28 (41)^*^	13 (17)^*^	33 (22)	29 (37)^*^
Angina (*n*, %)	33 (27)	20 (17)	11 (16)	16 (21)	35 (24)	12 (15)
Syncope (*n*, %)	19 (15)	10 (9)	12 (17)	13 (17)	16 (11)	12 (15)
Atrial fibrillation (*n*, %)	8 (6.5)^‡^^#^	23 (21)^#^	27 (39)^‡^	3 (3.9)^*^^‡^	20 (13)^*^	35 (45)^‡^
Glomerular filtration rate <60 mL/min/1.73 m^2^	27 (22)^*^	25 (22)	25 (36)^*^	14 (18)^*^	35 (24)	27 (35)^*^
COPD (*n*, %)	49 (40)^*^	48 (43)	39 (57)^*^	33 (43)	58 (39)	45 (58)
Preoperative hemoglobin (g/dL) (*n* ± SD)	13.5 ± 1.3	13.1 ± 1.5	13.1 ± 1.5	13.7 ± 1.2	13 ± 1.4	13.1 ± 1.4
Body mass index > 30 Kg/m^2^ (*n*, %)	34 (27)	44 (40)	23 (33)	20 (26)	54 (36)	27 (35)
Body surface area (m^2^) (*n* ± SD)	1.76 ± 0.2	1.79 ± 0.2	1.77 ± 0.2	1.79 ± 0.2	1.76 ± 0.2	1.78 ± 0.2
**Left ventricle:**						
Peak aortic jet velocity (m/s) (*n* ± SD)	4.5 ± 0.6	4.5 ± 0.6	4.4 ± 0.7	4.5 ± 0.6	4.4 ± 0.6	4.5 ± 0.6
Mean gradient (mmHg) (*n* ± SD)	52 ± 14	54 ± 19	53 ± 15	52 ± 15	54 ± 17	53 ± 17
Indexed aortic valve area (cm^2^/m^2^) (*n* ± SD)	0.41 ± 0.11	0.42 ± 0.13	0.41 ± 0.13	0.41 ± 0.13	0.42 ± 0.13	0.40 ± 0.13
LV ejection fraction <50% (*n*, %)	9 (7.3)	9 (8.1)	11 (16)	5 (6.5)	12 (8.1)	12 (15)
**Right ventricle:**						
Right ventricular basal diameter (mm) (*n* ± SD)	29 ± 4^‡^	31 ± 5^‡^	34 ± 5^‡^	29 ± 4^‡^*^^	30 ± 4^*^	35 ± 6^‡^
TAPSE <17 mm (*n*, %)	2 (1.6)^‡^*^^	6 (5)^*^	12 (17)^‡^	1 (1.3)^‡^	5 (3.4)	14 (18)^‡^
Inferior vena cava (mm) (*n* ± SD)	8.2 ± 4.2^‡^	9.1 ± 4	12.1 ± 5^‡^	8.1 ± 4.0^‡^	9.0 ± 4.5	12 ± 4.6^‡^
**Surgical parameters:**						
Biological prosthesis valve (*n*, %)	88 (71)	82 (74)	54 (78)	56 (73)	107 (72)	61 (78)
Bypass (*n*, %)	41 (33)^*^	21 (19)^*^	19 (27)	24 (31)^*^	37 (25)^*^	20 (26)
**Clinical outcomes during follow-up:**						
30 days postoperative (*n*, %)	3 (2.4)	3 (2.7)	2 (2.9)	1 (1.3)	5 (3.2)	2 (2.6)
All-cause mortality (*n*, %)	41 (33)^*^	39 (35)	35 (51)^*^	21 (27)^*^	54 (36)	40 (51)^*^

* *p < 0.05 individual category vs. low group*.

# *p = 0.001 individual category vs. low group*.

‡ *p <0.001 individual category vs. low group*.

Regarding the low PH probability group, 106 of the 124 patients of the low group with the guidelines threshold did not present any sign of right cavities involvement, and of these, 32 (30%) died. With the new cut-off point, 67 of the 77 patients of the low group did not show any right-side involvement sign, and of these, 17 (25%) patients died. No significant differences were observed between the three groups when comparing prosthetic mean gradient, 30 days and 3 months after surgery.

**Figure 2:**
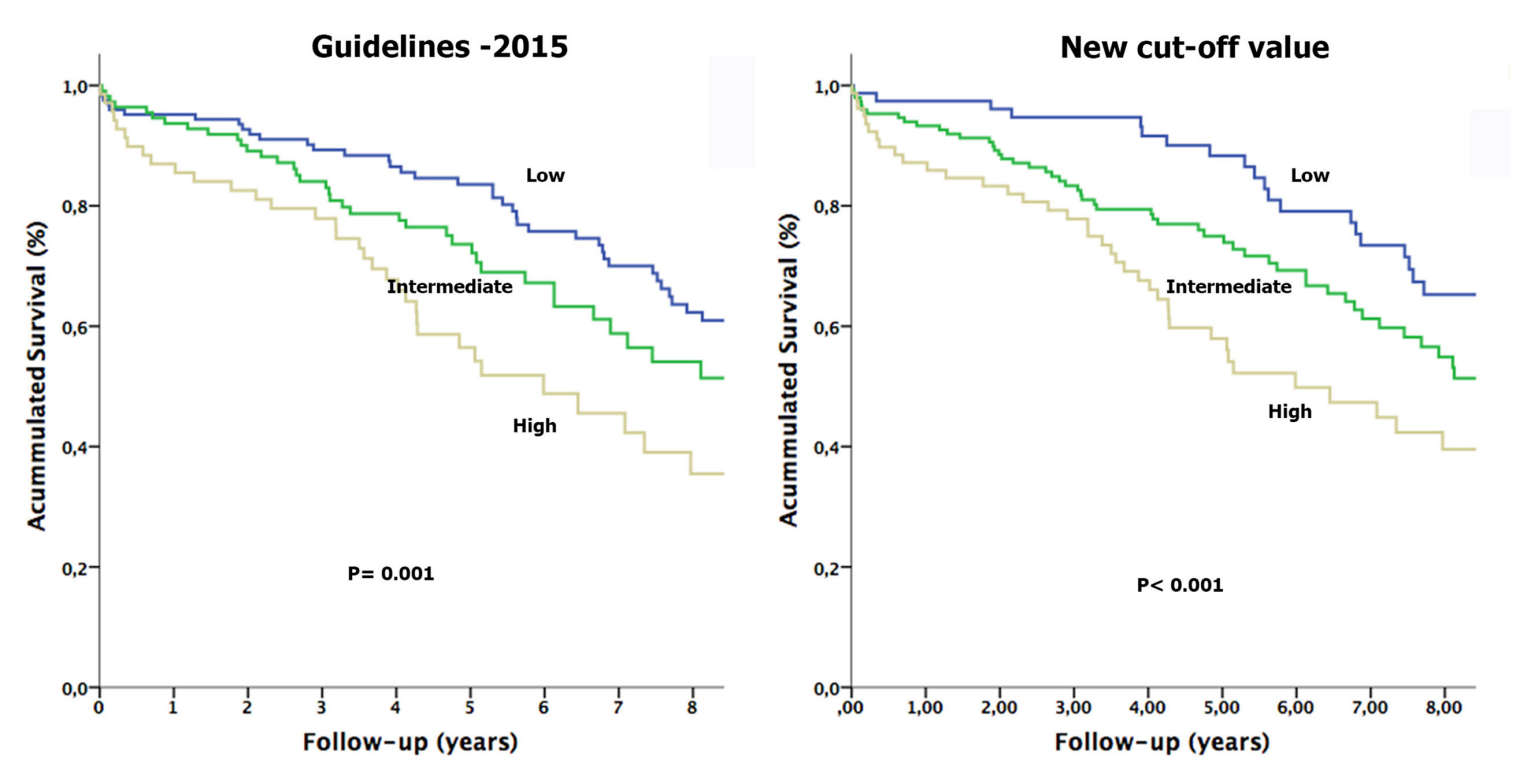
Kaplan-Meier survival curves analysis of patients with tricuspid regurgitation and an intermediate pulmonary hypertension probability compared to the low group, according to ESC/ERS guidelines and the new cut-off point thresholds.

The Kaplan Meier curves to time-to-event data (Figure 2) showed that the intermediate group mortality with the guidelines threshold did not significantly differ from the low group (Log-rank *p* = 0.86). Conversely, the intermediate group with the new cut-off point showed a significantly worse survival, compared to the low group (Figure 2, Log-rank *p* = 0.02). When performing the multivariate analysis, after adjusting for age, sex, and significant variables related to PH and mortality, the intermediate group with the new cut-off point remained independently associated with an increased overall mortality risk (HR 2.04; 95% CI: 1.91 to 3.48, *p* = 0.01), in contrast to the intermediate group with the guidelines threshold (HR 1.44; 95% CI: 0.89 to 2.32, *p* = 0.14; Table 3).

**Table 3:** Relative risk of all-cause mortality on multivariate analysis according to ESC/ERS and the new cut-off point thresholds, in patients with tricuspid regurgitation.

	**Guidelines−2015**			**New cut-off point**		
	**HR**	**CI**	**P**	**HR**	**CI**	***P***
Low PH probability group	Reference group			Reference group		
Intermediate PH probability group	1.44	0.89–2.32	0.14	2.04	1.91–3.48	0.009
High PH probability group	1.64	0.98–2.74	0.05	2.02	1.10–3.72	0.02

*Adjusted by age, sex, atrial fibrillation, COPD, left ventricular ejection fraction <50%, hypertension, diabetes mellitus, NYHA III-IV class, glomerular filtration rate <60 mL/min/1.73 m^2^, bypass, prosthesic valve*.

### Baseline Characteristics and Clinical Outcomes With all Patients

Baseline characteristics of all patients (444 patients) divided into three groups did not significantly vary from TRV patients' analyses (Supplementary Table 1).

TR was classified as non-measurable in 140 patients, and of these, 49 (35%) died during follow-up. 8 patients presented two echocardiographic signs of right chambers involvement, and of these, 6 (75%) died. The remaining 132 patients showed none or only one echocardiographic sign, and of these, 43 (33%, *p* = 0.02) died. Likewise patients with TR, no significant differences were observed between the three groups when comparing prosthetic mean gradient, 30 days and 3 months after surgery.

Similarly to the inclusion of patients with TR, the Kaplan-Meier survival curves correctly classified the three groups for both cut-off points (Log-Rank *p* < 0.001; Figure 1). When performing the multivariate analysis, the intermediate group obtained for both cut-off points remained an independent risk factor with an increased overall mortality compared to the low group: HR 1.62 (95% CI: 1.11–2.35, *p* = 0.01) for the new cut-off point; and HR 1.43 (95% CI: 0.96–2.13, *p* = 0.07) for guidelines threshold (Table 4).

**Table 4:** Relative risk of all-cause mortality on multivariate analysis according to ESC/ERS and the new cut-off point thresholds, in all patients.

	**Guidelines−2015**			**New cut-off point**		
	**HR**	**CI**	**P**	**HR**	**CI**	***P***
Low PH probability group	Reference group			Reference group		
Intermediate PH probability group	1.43	0.96–2.13	0.07	1.62	1.11–2.35	0.01
High PH probability group	1.46	0.94–2.29	0.09	1.51	0.94–2.40	0.08

*Adjusted by age, sex, atrial fibrillation, COPD, left ventricular, ejection fraction <50%, hypertension, diabetes mellitus, NYHA III-IV class, glomerular filtration rate < 60 mL/min/1.73 m^2^, bypass, prosthesis valve*.

### Correlation Between TRV Peak and Additional Echocardiographic Signs

**Figure 3:**
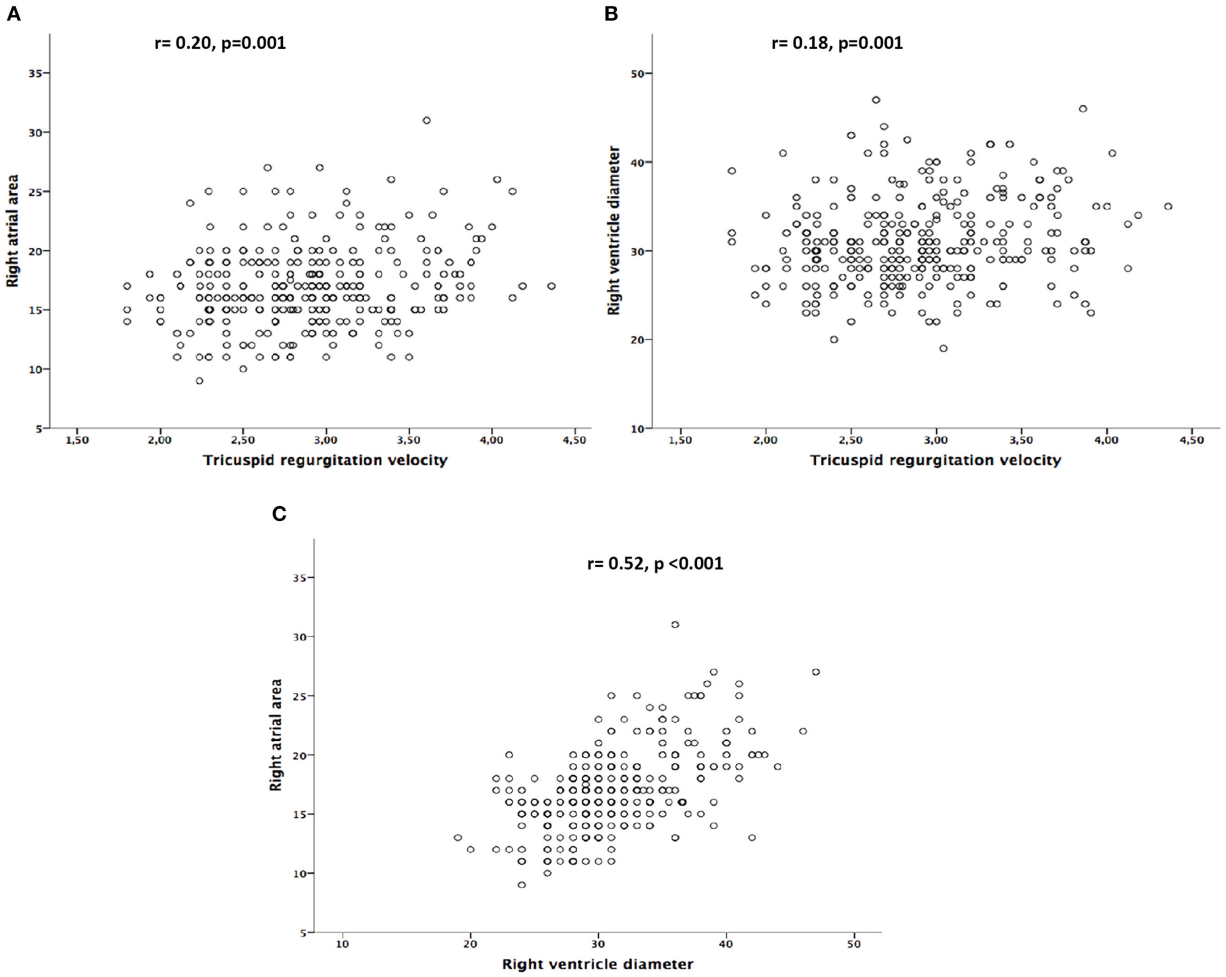
**(A)** Correlation between tricuspid regurgitation velocity and right atrial area. **(B)** Correlation between tricuspid regurgitation velocity and right ventricle diameter. **(C)** Correlation between right atrial area and right ventricle diameter.

Globally, TRV showed a weak association with RAA (*r* = 0.20, *p* = 0.001) and RV diameter (*r* = 0.18, *p* = 0.001). Conversely, a greater association was found between RAA size and RV diameter (*r* = 0.52, *p* < 0.001; Figure 3). When TRV was divided according to TR severity degree, the association between TRV and RAA remained weak (*r* = 0.14, *p* = 0.04) in mild TR, increasing up to *r* = 0.40 (*p* < 0.001) in moderate TR. Regarding RV diameter, while no association with TRV was found in mild TR, a moderate correlation was observed (*r* = 0.38, *p* = 0.001) in moderate TR.

## Discussion

The main findings of this observational study are the following: (1) A TRV cut-off point of 2.55 m/s, instead of the 2.8 m/s threshold proposed by the ESC/ERS guidelines, together with the measurements of the right cavities, allowed a better risk assessment of patients with PH secondary to severe aortic stenosis, with or without TR. (2) The association between right cavities size and TRV peak was weak, especially in mild TR.

Prior studies showed that a high PH probability was associated with an increased morbidity and mortality in patients with severe aortic stenosis (8–10). However, data are not so conclusive when it comes to lower velocities and the physiological TRV spectrum for healthy individuals remains controversial. The current guidelines suggest that PH probability is unlikely if TRV is <2.8 m/s and none or only one additional PH sign is present. Conversely, Marra et al. (6) observed that the 95% quantile of TRV in a healthy population corresponded to 2.55 m/s, without significant differences found between males and females. Moreover, in the Gladwin et al. study (4), a TRV ≥ 2.55 m/s was associated with a higher risk of death in patients with sickle cell disease; and Hachulla et al. (3) concluded that a TRV ≥2.55 m/s, together with symptoms of PH, effectively detected the disease at an early stage. Finally, in a large study that included 47,728 patients, Huston et al. (5) observed that when taking a TRV value of 1.9 m/s as reference, the mortality risk began at a TRV value of 2.3 m/s (HR 1.14; 95% CI 1.02–1.27) and doubled at a TRV value of 2.8 m/s (HR 2.08; 95% CI 1.83–2.37).

In the present study, the intermediate group with a TRV > 2.55 m/s was independently associated with a higher overall mortality risk (HR 2.04; 95% CI: 1.91–3.48, *p* = 0.01) compared to the low probability group. In contrast, the multivariate analysis for the intermediate group with TRV > 2.8 m/s did not reach significance (HR 1.44; 95% CI: 0.89–2.32, *p* = 0.14; Table 3). These findings thus suggest that the current recommended TRV threshold of 2.8 m/s might be missing an at-risk population, in the line with the emerging data that associated borderline PH with an increased mortality risk (13–16).

A weak association was observed between right cavities size and TRV (*r* = 0.20, *p* = 0.001 for RAA; and *r* = 0.18, *p* = 0.001 for RV diameter), especially in mild TR (Figure 3). These results were in accordance with prior studies that demonstrated that structural right- side changes, as right atrium enlargement and tricuspid annulus dilatation, could be present despite mild TR degree (17, 18). The low correlation found between TRV and right cavities measures may be explained by the suboptimal sensitivity and specificity of the TR jet used in isolation. This low association was already described in previous reports that compared PH estimation through the TR jet with right-heart catheterization, as the gold standard diagnostic technique (19, 20). In line with this concern, O'Leary et al. (21) showed, in a large study of 1,262 patients, that the lack of measurable TRV could not be equated with significant PH absence, as 47% of the patients without TR had confirmed PH by right heart catheterization. On another hand, other authors observed that patients with mild PH (5) and those in whom pulmonary artery pressure was underestimated by Doppler echocardiography (19) had evidence of RV dysfunction and dilation. In this regard, Mutlak et al. (22) observed that although pulmonary artery systolic pressure was a strong determinant of TR severity, others aspects such as demographic characteristics, mechanical factors or remodeling of the right heart cavities were also predictive of the TR jet, and that systolic pulmonary artery pressure was not always related to TR severity.

The current study also aimed to investigate the PH prognosis of those patients without TR, a less evaluated group due to its frequent exclusion in the majority of studies. Of the 140 patients with non-measurable TR, 75% (6 of 8 patients) with enlarged right chambers died during follow-up. Conversely, of the 132 remaining patients (with none or only one PH echocardiographic sign), 43 patients (33%, *p* = 0.02) died. When including all patients, the intermediate group obtained for both cut-off points remained an independent risk factor with an increased overall mortality compared to the low group: HR 1.62 (95% CI 1.11 to 2.35 *p* = 0.01) for the new cut-off point; and HR 1.43 (95% CI: 0.96 to 2.13, *p* = 0.07) for guidelines threshold (Table 4).

Secondary TR is caused by RV elliptical deformation and dysfunction, leading to valvular tethering and malcoaptation, and frequently only with mild tricuspid annulus enlargement (23–27). These anatomical changes were described in the absence of substantial TR (23, 26). Our findings may therefore be related to the different RV remodeling patterns in the PH setting, and might indicate that right cavities enlargement was strongly associated with worse outcomes. In this regard, previous studies demonstrated the importance of right cavities size in PH prognosis assessment (28–33). On the other hand, since the right ventricular muscle mass is lower than the one on the left ventricle, this makes RV systolic function more load sensitive. Consequently, TR severity relies on dynamic processes as preload, afterload and RV function (23, 25, 26), making TR grading extremely labile. Moreover, TR evaluation is echocardiographic window and respiratory dependent, leading to PH underestimation when the regurgitant jet is not easily recorded (17). These findings thus emphasized the need to use additional PH echocardiographic features as right-side measurements, particularly in those groups with mild or absent TR.

## Limitations

The current study had several limitations. First, this was a single-center observational study and therefore may not reflect general practice. All data were collected prospectively, although the analysis of the data was done retrospectively. Second, our findings concerning pulmonary pressure estimation were not compared with right heart catheterization measures. Third, no echocardiographic contrast had been used to potentiate TR in those patients with weak signal. Fourth, only two echocardiographic signs of different categories have been evaluated, easily obtainable in clinical practice, without meaning to compare the different echocardiographic signs proposed in ESC/ERS guidelines.

## Conclusions

A TRV cut-off point of 2.55 m/s, instead of the 2.8 m/s threshold suggested by the current guidelines, together with additional signs of right cavities involvement, provided a better risk stratification of PH secondary to severe aortic stenosis, with or without TR. Further prospective studies are warranted for confirmation and validation.

## Data Availability Statement

The original contributions presented in the study are included in the article/Supplementary Material, further inquiries can be directed to the corresponding author.

## Ethics Statement

The studies involving human participants were reviewed and approved by CEIm-Parc de Salut Mar institution (approval number 2019/8278). The Ethics Committee waived the requirement of written informed consent for participation.

## Author Contributions

CG-R and MC: conceptualization, data curation, formal analysis, methodology, validation, writing, review, and editing. MB, MG, AM-S, and NF-L: data curation, formal analysis, and writing. All authors contributed to the article and approved the submitted version.

## Conflict of Interest

The authors declare that the research was conducted in the absence of any commercial or financial relationships that could be construed as a potential conflict of interest.

## Publisher's Note

All claims expressed in this article are solely those of the authors and do not necessarily represent those of their affiliated organizations, or those of the publisher, the editors and the reviewers. Any product that may be evaluated in this article, or claim that may be made by its manufacturer, is not guaranteed or endorsed by the publisher.
