# Supplementary material for: Importance of Tricuspid Regurgitation Velocity Threshold in Risk Assessment of Pulmonary Hypertension-Long-Term Outcome of Patients Submitted to Aortic Valve Replacement
Source: Front Cardiovasc Med. 2021 Nov 10;8:720643. doi: 10.3389/fcvm.2021.720643 (PMC8631497; doi:10.3389/fcvm.2021.720643)
Supplement: Supplementary file 1 [file Table_1.docx]

**Table 1 supplement. Baseline characteristics and all-cause mortality during follow-up between groups, according to current ESC/ERS guidelines and the new cut-off point thresholds, in all patients**

|  | **ESC/ERS guidelines - 2015** | | | **New cut - off point value** | | |
| --- | --- | --- | --- | --- | --- | --- |
|  | **Low** | **Intermediate** |  | **Low** | **Intermediate** |  |
|  | (n=256) | (n=119) | **High** | (n=209) | (n=157) | **High** |
|  |  |  | (n=69) |  |  | (n=78) |
| **Risk factors:** |  |  |  |  |  |  |
| Age (n±SD) | 72 ± 9 * | 73 ± 8 | 75 ± 7 * | 72 ± 9 * | 73 ± 9 | 76 ± 7 * |
| Male (n, %) | 149 (58) * | 53 (45) * | 41 (59) | 127 (61) * | 70 (45) * | 46 (59) |
| Hypertension (n, %) | 181 (72) | 90 (76) | 54 (78) | 145 (69) | 120 (76) | 60 (77) |
| Diabetes mellitus (n, %) | 75 (30) | 37 (31) | 24 (35) | 64 (31) | 47 (30) | 25 (32) |
| Dyslipidemia (n, %) | 151 (59) | 61 (51) | 38 (55) | 119 (57) | 90 (57) | 41 (53) |
| Current Smoking (n,%) | 31 (12) | 15 (13) | 6 (8.7) | 26 (12) | 11 (11) | 9 (12) |
| NYHA III-IV class (n, %) | 48 (19) ‡ | 22 (18) | 28 (41) ‡ | 35 (17) ‡ | 34 (22) | 29 (37) ‡ |
| Angina (n,%) | 69 (27) | 20 (17) | 11 (16) | 52 (25) | 36 (23) | 12 (15) |
| Syncope (n,%) | 39 (15) | 10 (8.4) | 12 (17) | 33 (16) | 126(10) | 12 (15) |
| Atrial fibrillation (n,%) | 12 (4.7) ‡ | 25 (21) ‡ | 27 (39) ‡ | 7 (3.3) ‡ | 22 (14) ‡ | 35 (45) ‡ |
| Glomerular filtration rate <60 mL/min/1.73 m^2^ | 54 (21) * | 25 (21) | 25 (36) * | 41 (20) * | 36 (23) | 27 (35) * |
| COPD (n,%) | 100 (39) * | 52 (44) | 39 (57) * | 84(40) * | 62 (40) | 45 (58) * |
| Preoperative haemoglobin (g/dL) (n±SD) | 13.6 ± 1.3 | 13.1 ± 1.5 | 13.1 ± 1.5 | 13.7 ± 1.3 | 13 ± 1.4 | 13.1 ± 1.4 |
| Body mass index >30 Kg/m^2^ (n,%) | 80 (31) | 48 (40) | 23 (33) | 66 (32) | 58 (37) | 27 (35) |
| Body surface area (m^2^) (n±SD) | 1.78 ± 0.2 | 1.79 ± 0.2 | 1.77 ± 0.2 | 1.79 ± 0.2 | 1.77 ± 0.2 | 1.78 ± 0.2 |
| **Echocardiography:** |  |  |  |  |  |  |
| **Left ventricle:** |  |  |  |  |  |  |
| Peak aortic jet velocity (m/s) (n±SD) | 4.5± 0.6 | 4.4± 0.6 | 4.4± 0.7 | 4.5± 0.6 | 4.4± 0.6 | 4.4± 0.6 |
| Mean gradient mmHg) (n±SD) | 53 ± 14 | 54 ± 19 | 53 ± 15 | 53 ± 15 | 53 ± 17 | 53 ± 17 |
| Indexed aortic valve area (cm^2^/m^2^) (n±SD) | 0.40 ± 0.13 | 0.42 ± 0.13 | 0.41 ± 0.13 | 0.41 ± 0.13 | 0.42 ± 0.13 | 0.40 ± 0.13 |
| LV ejection fraction <50% (n,%) | 15 (5.9) * | 10 (8.4) | 11 (16) * | 11 (5.3) * | 13 (8.3) | 12 (15) * |
| **Right ventricle:** |  |  |  |  |  |  |
| Right ventricular basal diameter (mm) (n±SD) | 29 ± 4 ‡ | 31 ± 5 ‡ | 34 ± 5 ‡ | 29 ± 4 ‡ * | 30 ± 4 * | 35 ± 6 ‡ |
| TAPSE <17mm (n, %) | 2 (0.8) ‡ * | 6 (5) * | 12 (17) ‡ | 1 (0.5) ‡ | 5 (3.2) | 14 (18) ‡ |
| Inferior vena cava (mm) (n±SD) | 8.3 ± 4.2 ‡ | 9.0 ± 4 | 12.1 ± 5 ‡ | 8.4 ± 4.0 ‡ | 9.0 ± 4.5 | 12 ± 4.6 ‡ |
| **Surgical parameters:** |  |  |  |  |  |  |
| Biological prosthesis valve (n,%) | 182 (71) | 87 (73) | 54 (78) | 150 (72) | 112 (72) | 61 (78) |
| Small prothesis valve (nº 19 and 21) (n,%) | 148 (61) | 70 (65) | 35 (57) | 119 (61) | 93 (64) | 41 (59) |
| Bypass (n,%) | 84 (33) * | 26 (22) * | 19 (27) | 67 (32) * | 42 (27) * | 20(26) |
| **Clinical outcomes during follow-up:** |  |  |  |  |  |  |
| 30 days postoperative (n, %) | 6 (2.3) | 3 (2.5) | 2 (2.9) | 4 (1.9) | 5 (3.2) | 2 (2.6) |
| 30 days prothesic mean gradient (mmHg) (n±SD) | 17 ± 8 | 18 ± 8 | 18 ± 8 | 16 ± 6 | 16 ± 7 | 15 ± 6 |
| 3 monts prothesic mean gradient (mmHg) (n±SD) | 18 ± 8 | 18 ± 8 | 17 ± 8 | 16 ± 6 | 16 ± 7 | 15 ± 6 |
| All-cause mortality (n, %) | 84 (33) * | 45 (38) | 35 (51) * | 64 (31) # | 60 (38) | 40 (51) # |

* p<0.05 individual category vs. Low group
# p=0.001 individual category vs. Low group
‡ p<0.001 individual category vs. Low group
