# Supplementary material for: Importance of Tricuspid Regurgitation Velocity Threshold in Risk Assessment of Pulmonary Hypertension-Long-Term Outcome of Patients Submitted to Aortic Valve Replacement
Source: Front Cardiovasc Med. 2021 Nov 10;8:720643. doi: 10.3389/fcvm.2021.720643 (PMC8631497; doi:10.3389/fcvm.2021.720643)
Supplement: Supplementary file 2 [file Table_2.docx]

Table 2 supplement. **Parameters associated with mortality on univariate analysis for all patients and for patients with tricuspid regurgitation.**

|  | **All patients** | | | **Patients with tricuspid regurgitation** | | |
| --- | --- | --- | --- | --- | --- | --- |
|  | Alive (n=280) | Dead (n=164) | P | Alive (n=189) | Dead (n=115) | P |
| **Risk factors:** |  |  |  |  |  |  |
| Age (n+A6:D7±SD) | 71 ± 9 | 75 ± 7 | <0.001 | 72 ± 9 | 76 ± 7 | <0.001 |
| Male (n, %) | 142 (51) | 101 (62) | 0.026 | 90 (48) | 68 (59) | 0.051 |
| Hypertension (n, %) | 191 (68%) | 134 (82) | 0.002 | 128 (68) | 95 (83) | 0.004 |
| Diabetes mellitus (n, %) | 70 (25) | 66 (40) | 0.001 | 42 (22) | 48 (42) | <0.001 |
| Dyslipidemia (n, %) | 151 (54) | 99 (60) | 0.187 | 101 (53) | 74 (64) | 0.062 |
| Current Smoking (n, %) | 33 (12) | 19 (12) | 0.949 | 16 (8.5) | 13 (11) | 0.414 |
| NYHA III-IV class (n, %) | 44 (16) | 54 (33) | <0.001 | 32 (17) | 43 (37) | <0.001 |
| Angina (n, %) 0.03 | 58 (21) | (42 (26) | 0.233 | 34 (18) | 29 (25) | 0.132 |
| Syncope (n, %) | 41 (15) | 20 (12) | 0.470 | 29 (15) | 12 (10) | 0.224 |
| Atrial fibrillation (n, %) | 33 (12) | 31 (19( | 0.039 | 29 (15) | 29 (25) | 0.034 |
| Glomerular filtration rate < 60 mL/min/1.73 m2 | 46 (16) | 58 (35) | <0.001 | 35 (19) | 41 (36) | 0.001 |
| COPD (n, %) | 104 (37) | 87 (53) | 0.001 | 75 (40) | 61 (53) | 0.023 |
| Preoperative haemoglobin (g/dL) (n±SD) | 13.5 ± 1.4 | 13.3 ± 1.4 | 0.660 | 13.3 ± 1.4 | 13.2 ± 1.5 | 0.461 |
| Body mass index > 30 Kg/m2 (n, %) | 97 (35) | 54 (33) | 0.713 | 62 (33) | 39 (33) | 0.842 |
| Body surface area m2 (n±SD) | 1.78 ± 0.19 | 1.78 ± 0.19 | 0.948 | 1.77 ± 0.19 | 1.77 ± 0.19 | 0.867 |
| **Left ventricle:** |  |  |  |  |  |  |
| Peak aortic jet velocity (m/s) (n±SD) | 4.5 ± 0.57 | 4.4 ± 0.59 | 0.601 | 4.5 ± 0.6 | 4.4 ± 0.6 | 0.100 |
| Mean gradient (mmHg) (n±SD) | 54 ± 16 | 53 ± 15 | 0.718 | 55 ± 16 | 53 ± 17 | 0.529 |
| Indexed aortic valve area (cm2/m2) (n±SD) | 0.41 ± 0.13 | 0,42 ± 0.11 | 0.709 | 0.41 ± 0.12 | 0.41 ± 0.11 | 0.907 |
| LV ejection fraction < 50% (n, %) | 15 (5.4) | 21 (13) | 0.006 | 13 (6.9) | 16 (14) | 0.043 |
| **Right ventricle:** |  |  |  |  |  |  |
| Right ventricular basal diameter (mm) (n±SD) | 29 ± 5 | 31 ± 5 | 0.03 | 31 ± 5 | 32 ± 5 | 0.026 |
| TAPSE <17 mm (n, %) | 17 (4.3) | 8 (4.9) | 0.771 | 12 (6.3) | 8 (7) | 0.836 |
| Inferior vena cava (mm) (n±SD) | 9 ± 4 | 9 ± 5 | 0.338 | 9 ± 4 | 10 ± 5 | 0.295 |
| **Surgical parameters:** |  |  |  |  |  |  |
| Biological prosthesis valve (n, %) | 179 (64) | 144 (88) | <0.001 | 122 (65) | 102 (89) | <0.001 |
| Bypass (n, %) | 67 (24) | 62 (38) | 0.002 | 39 (21) | 37) | 0.002 |
|  |  |  |  |  |  |  |
| **ESC/ERS Guidelines - 2015** |  |  |  |  |  |  |
| Low | 172 (62) | 84 (52) |  | 83 (44) | 41 (36) |  |
| Intermediate | 74 (26) | 45 (27) |  | 72 (38) | 39 (34) |  |
| High | 34 (12) | 35 (21) | 0.023 | 34 (18) | 35 (30 | 0.040 |
| **New cut-off point** |  |  |  |  |  |  |
| Low | 145 (52) | 64 (39) |  | 56 (30) | 21 (18) |  |
| Intermediate | 97 (34) | 60 (37) |  | 95 (50) | 54 (47) |  |
| High | 38 (14) | 40 (24) | 0.005 | 38 (20) | 40 (35) | 0.007 |
