# Supplementary figures and images for: Importance of Tricuspid Regurgitation Velocity Threshold in Risk Assessment of Pulmonary Hypertension-Long-Term Outcome of Patients Submitted to Aortic Valve Replacement
Source: Front Cardiovasc Med. 2021 Nov 10;8:720643. doi: 10.3389/fcvm.2021.720643 (PMC8631497; doi:10.3389/fcvm.2021.720643)

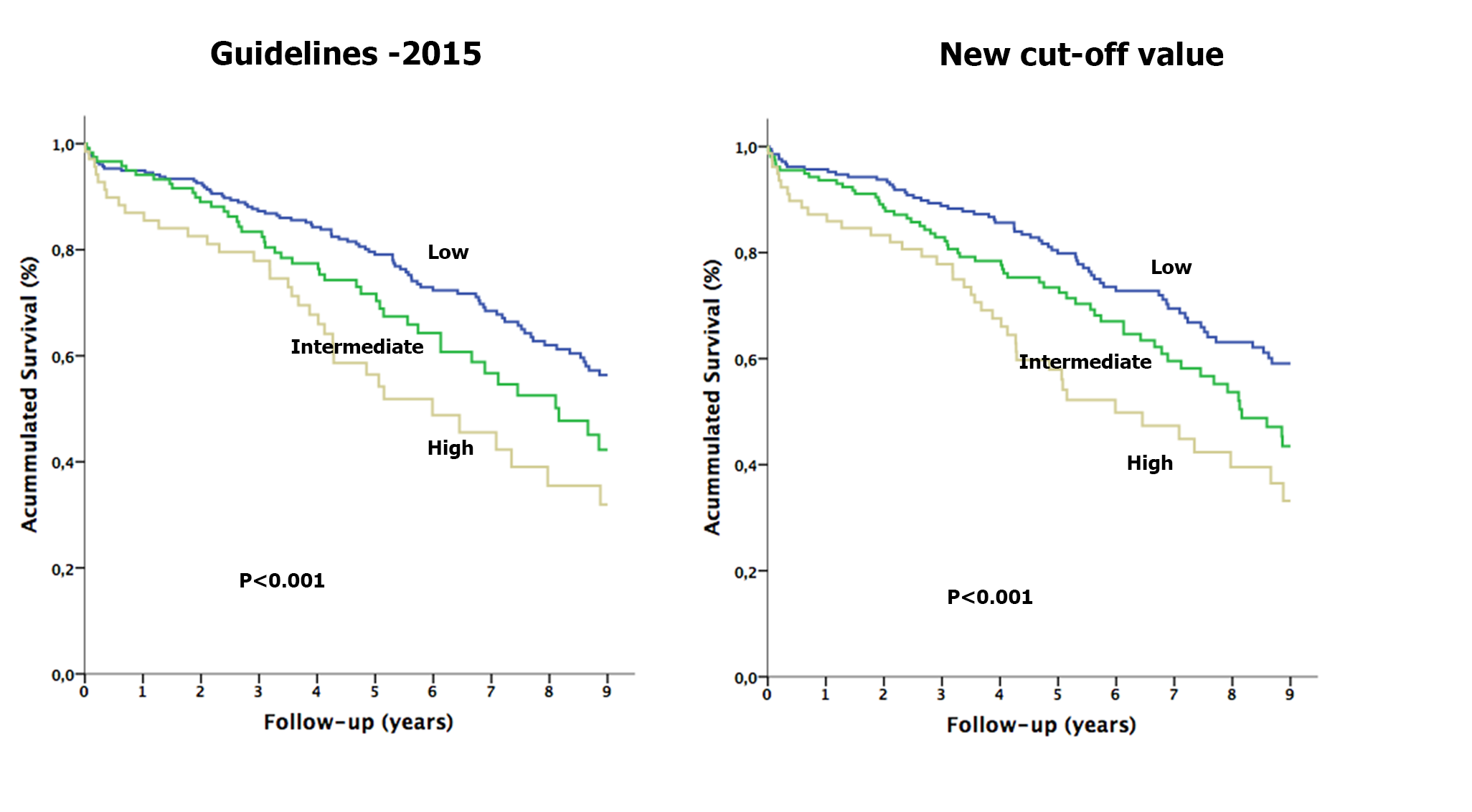

Supplement: Supplementary file 3 [file Image_1.TIF]
